# Study on Preparation of Regenerated Cellulose Fiber from Biomass Based on Mixed Solvents

**DOI:** 10.3390/ma17040819

**Published:** 2024-02-08

**Authors:** Junjiang Xiao, Pengcheng Li, Xiaotao Zhang, Ximing Wang

**Affiliations:** 1 College of Material Science and Art Design, Inner Mongolia Agricultural University, Hohhot 010018, China; 18469121639@163.com; 2 Central Grain Reserve Qingdao Depot Co., Ltd., Qingdao 266109, China; segkxnr@163.com; 3 College of Science, Inner Mongolia Agricultural University, Hohhot 010018, China; 4 Inner Mongolia Key Laboratory of Sandy Shrubs Fibrosis and Energy Development and Utilization, Hohhot 010018, China

**Keywords:** *Arundo donax* Linnaeus, cellulose fiber regenerated from biomass, TH/DS system, high-value utilization, antibacterial properties, antiviral properties

## Abstract

In this study, *Arundo donax* Linnaeus was utilized as the biomass and a TH/DS (Tetra-n-butylammonium hydroxide/Dimethyl sulfoxide, C_16_H_37_NO/C_2_H_6_OS) system was employed to dissolve biomass cellulose. The optimal process for the preparation of *Arundo donax* L. biomass regenerated cellulose fiber was determined through process optimization. The physical properties and antimicrobial performance of the resulting products were analyzed. The results demonstrated that the physical indicators of biomass regenerated cellulose fiber, prepared from *Arundo donax* L. cellulose, met the requirements of the standard for Viscose Filament (Dry breaking strength ≥ 1.65 CN/dtex, Elongation at dry breaking 15.5–26.0%, and Dry elongation CV value ≤ 10.0%). Additionally, excellent antimicrobial properties were exhibited by the biomass regenerated cellulose fiber developed in this study, with antibacterial rates against *Staphylococcus aureus* and other three strain indexes meeting the Viscose Filament standards. Furthermore, high antiviral activity of 99.99% against H1N1 and H3N2 strains of influenza A virus was observed in the experimental samples, indicating a remarkable antiviral effect. Valuable references for the comprehensive utilization of *Arundo donax* L. biomass resources are provided by this research.

## 1. Introduction

Currently, there is a global resource scarcity problem, compounded by limited domestic cotton production in China. Hence, the textile industry is urgently looking for biodegradable material and alternatives to scarce resources [1,2,3,4]. Since the widespread cultivation and planting of *Arundo donax* L. in China, it has become a crucial ecological barrier for environmental protection in the Yellow River Basin and Northwest Desert. *Arundo donax* L. belong to the Poaceae, Arundo, which have developed a rhizome, and are stout, erect and tough, with numerous nodes, often branched. *Arundo donax* L. like wet or cold conditions, such as in Guangdong, Xinjiang, etc. *Arundo donax* L., with young branches and leaves and with crude protein of up to 12%, is a good green feed for livestock.

*Arundo donax* L. has the advantages of high biomass, strong adaptability, high photosynthetic efficiency and fast growth. *Arundo donax* L. has a high adsorption capacity for heavy metals, such as cadmium and mercury, and can be used to repair contaminated soil. *Arundo donax* L. also has the advantage of high cellulose and crude protein content, which can be used in the production of fiberboard and chemical products (such as nano-fiber and xylose). *Arundo donax* L. can be widely used in ecological management, and can significantly increase the content of organic matter in sandy soil.

Compared with other plants, *Arundo donax* L. contains a high content of gramine and caranine, which are widely used in medicine and the chemical industry as known natural products. At present, gramine and its derivatives have good anti-tumor effects, and caranine has broad-spectrum bactericidal effects.

**Figure 1:**
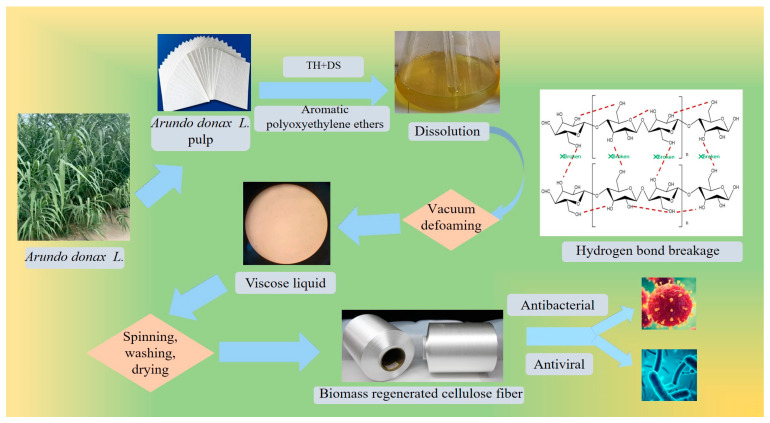
Biomass *Arundo donax* L. regenerated cellulose fiber.

In addition, *Arundo donax* L. has a more plentiful cellulose content, and is used to make high-quality pulp and rayon as a raw material. This has led to the biomass *Arundo donax* L. industry benefiting from abundant natural resources and other advantages [5]. Therefore, the efficient utilization of a large amount of *Arundo donax* L. has become a prominent concern in current textile research and development. However, this presents a difficult issue in the textile field. One particular challenge is the production of plant-based regenerative cellulose fiber using *Arundo donax* L. pulp as a new biomass material to replace cotton and wood pulp, and to manufacture biomass regenerated cellulose fiber instead from *Arundo donax* L. pulp. The flowchart for the preparation of regenerated cellulose fibers from biomass *Arundo donax* L. is shown in Figure 1.

This study investigates spinning process technology in the production of regenerated cellulose fiber from *Arundo donax* L. biomass using a novel solvent method. The study uses *Arundo donax* L. biomass cellulose and new environmentally friendly solvents as biomass [6,7,8,9,10]. Through continuous adjustment of the production process parameters for the regenerated cellulose fiber obtained from *Arundo donax* L. biomass, alongside improvement in the related technical indexes, the efficient preparation of this fiber using a novel solvent method was successfully achieved. The objective of this study is to nurture the development of the chemical fiber industry, while simultaneously achieving high value-added comprehensive recycling of *Arundo donax* L.’s high-quality resources. *Arundo donax* L. is a biomass that offers abundant yield, a low price, and a manufacturing process that is both green and clean. The industrial production of cellulose fiber regenerated from *Arundo donax* L. biomass can be achieved using this material. Such production has the potential to further advance China’s textile fiber industry and contribute to the country’s dual-carbon action plan [11,12,13].

## 2. Materials and Methods

### 2.1. Materials

*Arundo donax* L. (self-planted) cotton pulp was purchased from Xinjiang Egret FIBER Co., Ltd. (Xinjiang, China), COSMO wood pulp was purchased from COSMO, Inc. (Cosmopolis, WA, USA), broad-leaved wood pulp was purchased from South African Pulp and Paper Industries Co., Ltd. (Johannesburg, South Africa), 55% Tetrabutylammonium hydroxide was purchased from Alfa Aesar Co., Ltd. (TH), Tianjin, China), 99.9% dimethyl sulfoxide was purchased from Dongli Fine Chemical Co., Ltd. (DS), Shandong, China), and aromatic poly-oxyethylene ethers from Shenyang Haobo Industry Co., Ltd. (Shenyang, China), Sodium hydroxide and sulfate acid were purchased from Tianjin Windship Chemical Reagent Technology Co., Ltd. (Tianjin, China). Sodium sulfate and zinc sulfate were purchased from Xilong Chemical Co., Ltd. (Shantou, China). The values for the chemical composition of *Arundo donax* L. can be seen in Table 1.

### 2.2. Experimental Process Principle

Under the TH aqueous solution environment, the macromolecular chain of biomass cellulose broke. TH effectively eliminated the previous intermolecular hydrogen bonding in the process, which led to the reduction in biomass cellulose polymerization [14,15,16,17]. With the addition of DS, biomass cellulose was activated and quickly dissolved in the reagent with the assistance of the aromatic polyoxyethylene ether catalyst. The spinning gels were prepared initially through the process of gel filtration and static vacuum defoaming. Later, the operation of metering pump, coagulation bath, and spinning machine successfully prepared regenerated cellulose fiber from *Arundo donax* L. biomass.

### 2.3. All of Experimental Process Route

*Arundo donax* L. → *Arundo donax* L. pulp → dissolving reagent → stirring → dissolving → vacuum defoaming → viscose liquid → coagulation bath → spinning → washing and drying → biomass *Arundo donax* L. regenerated cellulose fiber.

**Figure 2:**
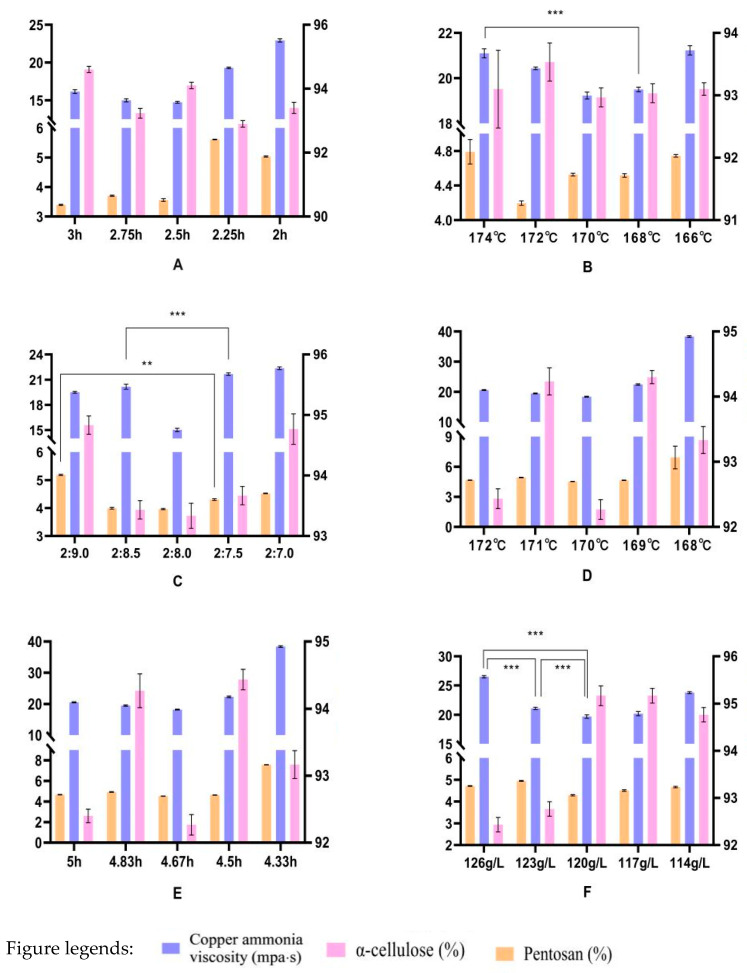
The experimental process parameters of biomass *Arundo donax* L. pulp. (**A**) Effect of steam time to pulp indexs; (**B**) Effect of steam temperature to pulp indexs; (**C**) Effect of the ratio of *Arundo donax* L.-alkali to pulp indexs; (**D**) Effect of the cook temperature to pulp indexs; (**E**) Effect of the cook time to pulp indexs; (**F**) Effect of the alkali concentration to pulp indexs. ** represent *p* < 0.05, *** represent *p* < 0.01.

*Arundo donax* L. materials cutted into rectangular, side length of 2~2.5 cm material, flowing water repeatedly washing *Arundo donax* L. mud scale, metal impurities, debris and other attachments, dry water. Making *Arundo donax* L. Pulp process, pretreatment process is mainly to analyze the steam time, steam temperature on the *Arundo donax* L. pulp impurity removal degree. The optimum value (Figure 2A–F) of each factor was deduced by discussing the important indexes of the size through different materials-alkali proportion, cooking temperature, cooking time and alkali concentration. The pulping process was carried out on the high temperature pulping material, the speed of the pulping machine was set at 8.0~8.5 r/s, the working temperature was 35 °C and the beating time was 0.5~1 h, press molding.

**Figure 3:**
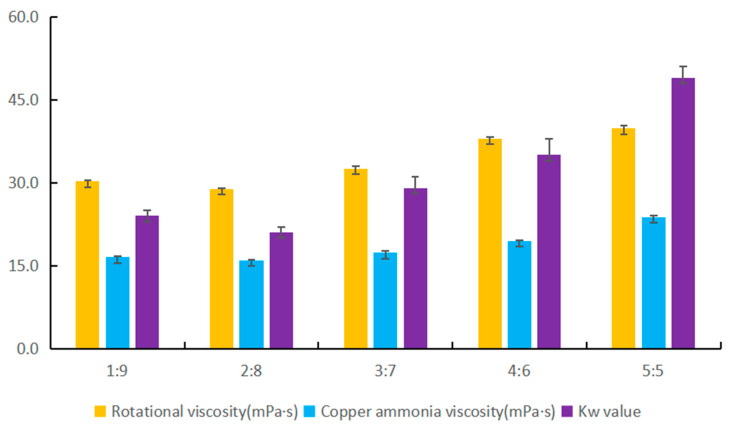
Analysis of the performance of the prepared *Arundo donax* L. cellulose viscose solution.

The Activation time, TH:DS solvent ratio, vacuum defoaming time and Aromatic polyoxyethylene ether dosage of different pulps were discussed. The influence of the above indexes on the quality of viscose liquid is analyzed in detail with Figure 3.

Coagulation bath: Sufficient sodium sulfate, with a content range of 50–300 g/L, was selected for the coagulation bath, and the acid bath was kept at a suitable temperature range of 25–50 °C and an acid concentration of 50–200 g/L. A minimum of 10.0–15.0 g/L zinc sulfate was added to the bath.

Spinning, washing and drying: The particular procedure entails controlling spinning speed between 50–150 m/min, using a spinning sizing amount ranging from 0.5 to 4.5%, and employing a spinning metering pump flow rate of 0.540–0.725 mL/r. Subsequently, the *Arundo donax* L. regenerated cellulose fiber that have been washed and dried are coiled on a tube.

### 2.4. Experimental Process Conditions

*Arundo donax* L. pulp:

After repeatedly cleaning and drying the sterilized biomass *Arundo donax* L., the *Arundo donax* L. grass is placed in a high-temperature and high-pressure cooking vessel, which is pretreated by steam medium, the steaming temperature was 160–169 °C; the holding time was 1–2 h; the catalyst and assistant were 0–0.4 g and 0–0.5 mL respectively. After removed impurities with desalted water, the pretreated *Arundo donax* L. was put into the cooking vessel, followed by adding concentrated alkali with the concentration of 105–120 g/L, adding 2–5 g catalyst and 1–5 mL auxiliary agent into the cooking vessel (the cooking temperature was 165–170 °C and the holding time was 4–6 h) [18,19,20]. The prepared pulp was reprocessed which the beating temperature was 30–35 °C; the beating time was 30–45 min; the final cycle was bleached and then the ash-removing iron was added, and the *Arundo donax* L. pulp was made.

Preparation of biomass cellulose viscose liquid:

To prepare a mixed solubilization system of TH and DS, the *Arundo donax* L. pulp was sterilized and dried using a 0.5–20.0% ethanol solution. Additionally, a specific amount of aromatic polyoxyethylene ether additives were included to promote the cellulose solubilization in the biomass. The TH solution made up 45–55 wt% (with the other components being aqueous solution) and accounted for 5–50% of the solubilization system. The mass fraction of the DS solution was 85–99%. 9% by weight (other components consist of an aqueous solution), constituting 50–95% of the dissolved system. To optimize the process, it is preferable that the TH solution has a mass fraction of 50–55 wt% (with the remaining components being aqueous solution), accounting for 5–30% of the dissolved system. The DS solution should have a mass fraction of 90–98 wt% (with the remaining components being aqueous solution), accounting for 55–85% of the dissolved system. Additionally, the mass fraction of the solution should be 45–55 wt% (with the remaining components being aqueous solution), accounting for 5–50% of the dissolved system. 55 to 85% of the dissolution system; the ratio of volume of additives to *Arundo donax* L. pulp mass is (1 to 6)/(1 to 10) mL/kg; the temperature for *Arundo donax* L. grass cellulose dissolution is between 30 °C and 45 °C.

Preparation of spinning coagulation bath:

The new solvent solution, prepared with *Arundo donax* L. biomass cellulose liquid, was formed through an acid-base reaction in the coagulation bath to produce regenerated *Arundo donax* L. cellulose fiber. Sufficient sodium sulfate, with a content range of 50–300 g/L, was selected for the coagulation bath, and the acid bath was kept at a suitable temperature range of 25–50 °C and an acid concentration of 50–200 g/L. A minimum of 10.0–15.0 g/L zinc sulfate was added to the bath.

Preparation of *Arundo donax* L. regenerated cellulose fiber:

The process of producing a new solvent and spinning *Arundo donax* L. regenerated cellulose fiber resulted in the successful production of *Arundo donax* L. biomass regenerated cellulose fiber that comply with product standards for the textile chemical fiber industry. The spinning process was optimized by adjusting the spinning speed and controlling the flow rate of desalinated water and other parameters. The particular procedure entails controlling spinning speed between 50–150 m/min, using a spinning sizing amount ranging from 0.5 to 4.5%, and employing a spinning metering pump flow rate of 0.540–0.725 mL/r. Subsequently, the regenerated cellulose fiber derived from *Arundo donax* L. biomass, that have been processed utilizing a novel solvent method, are coiled on a tube and stored in the warehouse.

To summarize, the viscose liquid’s alpha cellulose index ranged from 7.5% to 8.5%, and the drop ball viscosity (drop ball method) index ranged from 25 to 45 s. The process of spinning regenerated cellulose fiber is as follows: spinning speeds of 50–150 m/min, spinning sizing volumes of 0.5–4.5%, and metering pump flow rates of 0.540–0.725 mL/r.

### 2.5. Experimental Calculation Formula

#### 2.5.1. Viscosity Analysis of Cupric Ammonia

At 20 °C was used to determine the outflow time of cellulose cupric ammonia solution through a capillary viscometer. Based on these determinations and the known concentration of cellulose cupric-ammonia solution, the pulp cupric-ammonia viscosity was calculated by dynamic viscometer (mPa·s).

η=ρtK

ρ—The density of the copper-ammonia solution, g/mL (ρ = 0.97);

*t*—The outflow time of the sample solution, s;

*K*—Calibration factor of capillary viscometer.

#### 2.5.2. *α*-Cellulose Analysis

The pulp for viscose fiber was treated with 17.5% sodium hydroxide solution at 20 °C, then washed with 9.5% sodium hydroxide solution, and finally washed, dried and weighed with water to obtain the a cellulose content, expressed as a percentage.

The formula for calculating the percentage *X* is as follows:

$$X=\frac{\left(m_{1}-m\right)}{m_{2}(1-W)}\times 100\%$$

*X*—*α*-Cellulose content, %;

*m*—Quality of sand core crucible, g;

*m*_1_—Quality of sand core crucible and a type of cellulose, g;

*m*_2_—Weigh the sample mass for humidity balance, g;

*W*—The moisture content of the sample was analyzed after humidity balance, %.

#### 2.5.3. Analysis of Pentosan Content

By heating the pulp with 12% hydrochloric acid, the polysaccharide in the pulp (*α*-cellulose) was converted to furfural.

The content of pentose was obtained from the empirical formula by calculating the amount of furfural. The formulae for both are given below:

$$X_{1}=\frac{(V_{1}-V_{2})\times A\times c\times 500}{200m}\times 100$$

*X*_2_ = 1.375 *X*_1_

*X*_1_—Content of furfural, %;

*X*_2_—Content of pentose, %;

1.375—Conversion of furfural to pentose;

*A*—The amount of furfural equivalent to 1 mL 1 mol/L sodium thiosulfate standard solution, *A* (Tetrabromination) = 0.024;

*V*_1_—Consumption of standard sodium thiosulfate solution during blank test, mL;

*V*_2_—Consumption of standard sodium thiosulfate solution for titrating samples, mL;

*c*—Concentration of sodium thiosulfate standard solution, mol/L;

*m*—Quality of dry test sample, g.

#### 2.5.4. Reaction Performance Analysis

The pulp reacts with a certain amount of sodium hydroxide and carbon disulfide to form cellulose sulfonate. To determine the time difference of cellulose sulfonate solution passing through the same volume of filter hole successively.

### 2.6. Data Processing

Set up multiple sets of repeatable experiments, and the data were processed with Graphpad Prism 8 and SPSS 25.

## 3. Results and Discussion

### 3.1. Preparation and Analysis of Arundo donax L. Pulp

In the pulping process, the pretreatment of *Arundo donax* L. materials (steam treatment stage) is mainly to remove some impurities and lignin. After the pre-treated material undergoes the cooking stage, where alkali is used to extract *α*-cellulose and catalyst is used to reduce the degree of polymerization and the copper ammonia viscosity of the *Arundo donax* L. material itself.

When the steaming time was 2.5 h, the copper ammonia viscosity was lower, the *α*-cellulose was better in the solution system, the pentosan and *α*-cellulose index of pulp were better. When the steaming temperature was 170 °C, the copper ammonia viscosity of the *Arundo donax* L. pulp was the lowest, which was 19.15 mPa∙s. About factors steaming time and steaming temperature involved copper ammonia viscosity, pentosan and *α*-cellulose index could reference Figure 2A,B.

The ratio of *Arundo donax* L.-alkali was 2:8, the copper ammonia viscosity is the best, the pentosan is the lowest, and *α*-cellulose met the relevant requirements. When the cooking temperature was set at 170 °C, the copper ammonia viscosity of the *Arundo donax* L. pulp was lower, and the performance of pentosan and *α*-cellulose was normally, but the pulp was of better quality. About factors materials-alkali and cooking temperature involved copper ammonia viscosity, pentosan and *α*-cellulose index could reference Figure 2C,D.

When the cooking time was set at 4.67 h, the copper ammonia viscosity was lower, and the pentosan and *α*-cellulose could meet the experimental requirements. At 120 g/L of alkali concentration, the copper ammonia viscosity and pentosan were the lowest, and the value of *α*-cellulose was the highest. About factors cooking time and alkali concentration involved copper ammonia viscosity, pentosan and *α*-cellulose index could reference Figure 2E,F (** represent *p* < 0.05, *** represent *p* < 0.01).

At the steaming stage, the viscosity of cupric ammonia and the content of pentosan were the lowest, and the *α*-cellulose could meet the requirement of industry. The results show that the viscosity of cupric ammonia and the content of pentosan can be reduced by steaming treatment at suitable temperature and time. In the cooking stage, the highest content of a-cellulose was found in the pulp prepared with 120 g/L alkali, which indicated that the appropriate concentration of alkali had higher extraction rate of a-cellulose. The too high concentration of alkali could decompose a-cellulose, which the results showed that the content of a cellulose in the pulp was low.

On the appearance and handle of the *Arundo donax* L. pulp has more black impurities, but the pulp has more toughness and it belongs to medium-long cellulose fiber (Table 2). The quality of *Arundo donax* L. pulp was similar with American COSMO Wood Pulp. The content of *α*-cellulose in *Arundo donax* L. pulp is close to that of American COSMO pulp, but the brightness of *Arundo donax* L. pulp is lower than that of other two pulps, and it’s viscosity of cupric ammonia more lower than other pulps. The ash content of *Arundo donax* L. pulp is higher, and which is leave room for further to study.

The *α*-cellulose content of *Arundo donax* L. pulp is as high as 91.5%, which is equal to the highest level of American COSMO Wood Pulp. However, the content of cellulose in South African broad-leaved forest pulp was much higher than the above two types, showing certain advantages. The average moisture content of *Arundo donax* L. pulp was higher than that of other pulps (the standard value of average moisture content was ≤13.0%), so it was necessary to prolong the drying time or improve the drying temperature to reduce the moisture content of *Arundo donax* L. pulp. The ash and iron content of *Arundo donax* L. pulp is about twice as much as that of COSMO Pulp and South African broad-leaved wood pulp. In addition, the dust content of *Arundo donax* L. pulp was higher than that of American COSMO Pulp and South African broad-leaved wood pulp. Therefore, to verify the high impurity content of *Arundo donax* L. pulp, it is necessary to further improve the technology of removing impurities and improve the quality of *Arundo donax* L. pulp.

The Cupric ammonia viscosity of *Arundo donax* L. pulp is 8.4 mPa·s, which shows superiority in the preparation of glue solution by cellulose dissolution and is suitable for the requirement of glue solution viscosity. The alkali absorbency of *Arundo donax L*. pulp was 608.0%, which was much higher than the standard level of chemical fiber pulp (alkali absorbency ≥ 450.0%). The alkali absorption value mainly expresses the degree of alkali absorption when the pulp dissolves, and the higher the alkali absorption value, the faster the pulp dissolves. The content of pentose in *Arundo donax* L. pulps was 4.28%, which accorded with the requirement of chemical fiber pulp (pentose ≤ 5.0%). The reactivity of *Arundo donax* L. pulp was 15.5 s, which was much lower than the standard value of pulp (reactivity ≤ 250.0 s).

### 3.2. Analysis of Indexes of Cellulose Dissolution Process

The study examined the impact of the TH:DS pairing on the effectiveness of an experimentally prepared viscose solution used to dissolve *Arundo donax* L. cellulose pulp. The investigation included analyzing the ratios of dissolving reagents, the percentage of *Arundo donax* L. cellulose pulp, activation time, viscose solution defoaming time, and the presence of additives (Table 3). Through examining various reagent ratios, we conducted an examination of the essential parameters of the viscose liquor to analyze its performance, ultimately determining the corresponding index values [8].

### 3.3. Performance Analysis of Cellulose Spinning Viscose Liquid

The viscose liquid of cellulose is the key to produce regenerated cellulose fiber. The rotational viscosity and cupric-ammonia viscosity of the viscose liquid represent the viscosity degree of the prepared viscose liquid. If too dense, it will lead to poor fluidity of the viscose liquid, which is unfavorable to the long-distance transportation of the viscose liquid. If the viscosity of the viscose liquid is too low, which reaction viscose liquid contains less cellulose, affect the preparation of fiber molding. *Kw* value reflects the degree of cellulose solubility, of course, the smaller the *Kw* value represents the viscose liquid did not dissolve completely cellulose more less.

TH and DS do not produce pungent odors, such as hydrogen sulfide, in the process of dissolving the *Arundo donax* L. pulp. The best cellulose gel outcomes were achieved with an optimal reagent ratio of 2:8, exhibiting superior rotational and copper ammonia viscosity, and lower relative *Kw* value than other experiments in Figure 3. The use of an unsuitable reagent ratio of 1:9 was found to be suboptimal [16,21,22]. For information regarding the theoretical dissolution of cellulose from biomass by utilizing the novel, eco-friendly TH solvent paired with DS, please refer to the research findings laid out by Wenjiao Ge et al. The figures depicted above showcase how an effective dissolution effect is attained through the prepared cellulose viscose process when the TH percentage within the mixed TH/DS solution does not surpass 30%. This method can be utilized to produce regenerated cellulose fiber from biomass derived from *Arundo donax* L.

The above phenomena indicate that more DS should be added in the process of cellulose dissolution, so that the cellulose can be fully dissolved. The TH solution can be contact with the cellulose inside, and the cellulose molecule can be broken. If the DS solvent content is low, it will cause TH solvent slowly into the cellulose inside, resulting in partial cellulose not completely dissolved phenomenon.

### 3.4. Analysis of Solidification Bath Process

Sulfuric acid plays an essential role in the production of regenerated cellulose fiber. A high concentration of acid affects both the internal and external layers of these fiber, resulting in poor physical properties. On the other hand, an insufficient amount of acid in the coagulation bath considerably slows down the regeneration rate during spinning, causing difficulties in the spinning of regenerated cellulose fiber.

Furthermore, the temperature of the acid bath has a significant impact on the internal ion concentration of the system. If the temperature is too high, it can cause the spinning process to accelerate, making it essential to select an appropriate acid bath temperature to ensure process stability. The optimal conditions for the acid bath process are: sulfuric acid concentration ranging from 100 g/L to 150 g/L, acid bath temperature set at 45 °C to 50 °C, sodium sulfate content between 220 g/L and 280 g/L, and zinc sulfate concentration kept at 10.0 g/L to 12.0 g/L.

### 3.5. Physical Property Analysis of Regenerated Cellulose Fiber from Biomass Arundo donax L.

The physical properties of regenerated cellulose fiber, especially Elongation at dry break and dry breaking strength, were analyzed by comparing different proportions of TH and DS. Dry breaking strength is the maximum amount of force that can be applied to a break. Elongation at dry break is the length of of the extension about fiber when it breaks under the action of a force.

**Figure 4:**
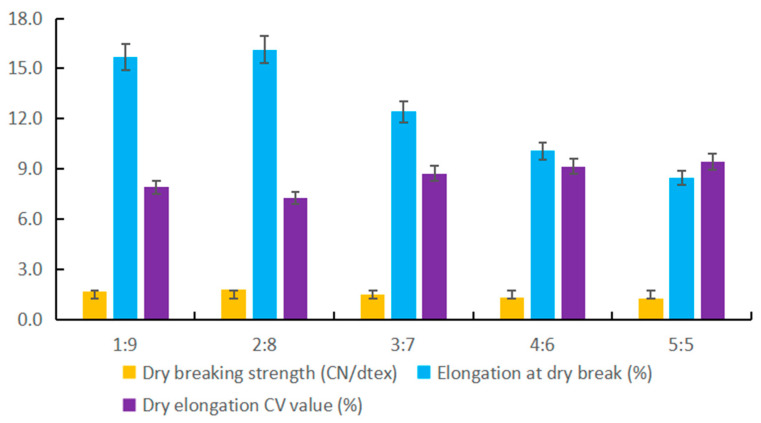
Physical properties of regenerated cellulose fiber (75D/40F) from biomass of *Arundo donax* L.

We conducted a study of the regenerated cellulose spinning experiment, accounting for 30% material ratio of TH. The research investigates physical properties of the regenerated cellulose fiber. Through experimentation, we obtained the performance indices of regenerated cellulose fiber from *Arundo donax* L. biomass. Specific data is presented in Figure 4 TH:DS = 2:8. The prepared biomass fiber exhibited a dry breaking strength of 1.77 CN/dtex, an elongation at dry breaking of 16.12%, and a dry elongation CV value of 7.26% under the environment of TH:DS. The findings suggest that the regenerated cellulose fiber prepared meets the standards of top-quality products, fulfilling the downstream producers’ requirement for biomass. Moreover, our review of pertinent literature reveals that the physical indices of the regenerated cellulose fiber sourced from *Arundo donax* L. biomass comply with the specifications (Dry breaking strength ≥ 1.65 CN/dtex, Elongation at dry breaking 15.5–26.0%, and Dry elongation CV value ≤ 10.0%) [23].

The above phenomena express the physical indexes of the regenerated cellulose fibers prepared with different proportions of TH and DS. The results showed that TH:DS = 2:8 was the best solvent for preparing cellulose viscose liquid, and the quality of cellulose viscose liquid was more better. It was also shown that low DS content would lead to poor physical properties of regenerated cellulose fibers. It is necessary to increase the ratio of DS to make cellulose fully swell in the process of viscose liquid preparation.

### 3.6. Analysis of Antimicrobial Properties of Biomass-Regenerated Cellulose Fiber from Arundo donax L.

*Arundo donax* L. contains large amounts of gramine, which has antibacterial properties. The antibacterial activity of regenerated cellulose fiber prepared from *Arundo donax* L. against *Escherichia coli* (ATCC 6538), *Staphylococcus aureus* (ATCC 10231) and *Candida albicans* (8099) were investigated. The antibacterial effect of *Arundo donax* L. cellulose fiber was analyzed by actual antibacterial value.

**Figure 5:**
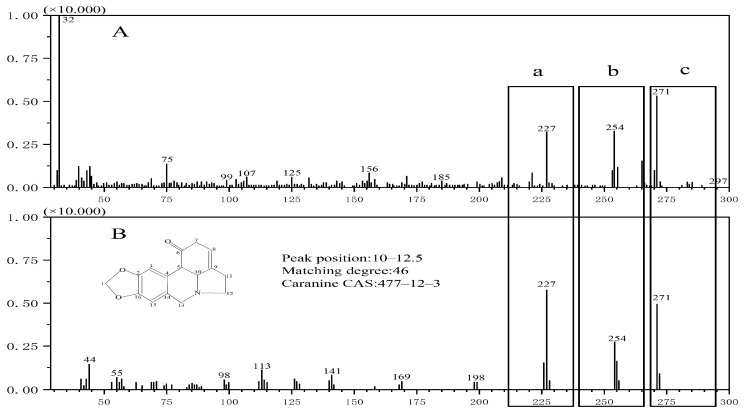
Qualitative mass spectrometry analysis of caranine. (**A**) shows the spectrum of *Arundo donax* L. fiber, and (**B**) shows the spectrum of the reference substance, caranine. (**a**) Formula is C_14_H_13_NO_2_ when the *m*/*z* of 227; (**b**) The *m*/*z* of 254 and formula is C_16_H_16_NO_2_; (**c**) The *m*/*z* of is 271 and the formula is C_16_H_17_NO_3_, which is the molecular weight of caranine.

Biomass cellulose fiber, derived from *Arundo donax* L. pulp, exhibits antimicrobial properties [24,25,26,27,28,29,30,31]. In accordance with evaluation of antimicrobial performance of textiles, the antimicrobial efficacy of cellulose fiber regenerated from biomass was assessed. As indicated by Table 4, the regenerated cellulose fiber that were prepared had outstanding antimicrobial properties against *Escherichia coli*, *Staphylococcus aureus*, and *Candida albicans*, and their measured values considerably exceeded the antimicrobial standards of production. This served as a commendable example of the utilization and production of new, functional eco-friendly materials. Qualitative analysis of the chemical composition of *Arundo donax* L. cellulose fiber revealed the presence of an antibacterial compound known as caranine (3,12-Didehydro-9,10-[methylenebis(oxy)]galanthan-1*α*-ol, refer to Figure 5). Additionally, caranine found in the fiber exhibits exceptional antibacterial properties [32,33,34,35].

The upper part (A) of Figure 5 shows the spectrum of molecular structure in the *Arundo donax* L. fiber, and the lower part (B) of Figure 5 shows the spectrum of the reference substance, caranine. Formula is C_14_H_13_NO_2_ when the *m*/*z* of Figure 5B(a) is 227. In the structural formula, carbon 5, carbon 6 and carbon 7, carbon 8 breaks and loses C_2_H_4_O. When the *m*/*z* of Figure 5B(b) is 254 and formula is C_16_H_16_NO_2_, C=O above carbon 6 in the structural formula breaks in the carbon chain and loses -OH. When the *m*/*z* of Figure 5B(c) is 271, the formula is C_16_H_17_NO_3_, which is the molecular weight of caranine. Figure 5A corresponds to Figure 5B, which can be inferred the Figure 5A represents caranine.

The Standard antibacterial value of *Escherichia coli* and *Staphylococcus aureus* is more 70%, 60% of *Candida albicans*. The results showed that the actual antibacterial level of *Arundo donax* L. fiber was more than 99%, and it had obvious antibacterial effect. The above situation may be inferred to the role of gramine inside the *Arundo donax* L.

The biomass fiber of *Arundo donax* L. possesses the following characteristics:

1. This plant fiber has natural antibacterial properties, with antibacterial components derived from internal biomass materials. These components have no negative effects; 2. The antibacterial effect lasts a long time due to the tight integration of the antibacterial components with natural plant cellulose. The finished product can be repeatedly washed and reused [36,37,38,39,40,41]; 3. The fiber is environmentally friendly as the biomass regeneration of the cellulose fiber can be fully degraded, and the preparation process is pollution-free. This is in compliance with green environmental protection requirements.

### 3.7. Analysis of Antiviral Properties of Biomass Regenerated Cellulose Fiber from Arundo donax L.

The regenerated cellulose fiber of *Arundo donax* L. contains caranine, which has antiviral properties. The antiviral activity of regenerated cellulose fiber prepared from *Arundo donax* L. against influenza A virus H3N2, influenza A virus H1N1 were investigated. The antiviral effect of *Arundo donax* L. cellulose fiber was analyzed by actual antiviral value.

The antivirals activity activity of an experimental sample was assessed using the ISO 18184:2019(E) standard method [42,43]. Results indicate that the regenerated cellulose fiber exhibits antiviral activity values greater than 4.85 and 99.99% antiviral activity against influenza A virus H3N2 (ATCC VR-1679) (Table 5). Similarly, regenerated cellulose fiber exhibits antiviral activity values greater than 5.05 and 99.99% antiviral activity against influenza A virus H1N1 (ATCC VR-1469). This study confirms that the regenerated cellulose fiber that were prepared demonstrate exceptional antiviral properties [44].

The results showed that the antivirus activity value of *Arundo donax* L. fiber was more than 4.85 of against influenza A virus H3N2, influenza A virus H1N1, and it had obvious antiviral effect. The above antiviral situation may be inferred to the role of caranine inside the *Arundo donax* L.

## 4. Conclusions

During the preparation of biomass cellulose pulp from *Arundo donax* L., the lignin and impurities in *Arundo donax* L. were removed by steaming. *Arundo donax* L. pulp proved that it was feasible to extract *α*-cellulose from *Arundo donax* L. by alkali to prepare pulp. However, the impurities in the pulp were found to be more higher than the normal pulp. Therefore, it is necessary continue to optimize the experimental process during the steaming stage of pretreatment and the washing pulp process. The prepared biomass fiber *Arundo donax* L. exhibited a dry breaking strength about 1.77 cn/dtex, and elongation at dry breaking about 16.12% when TH:DS = 2:8, which could meet the requirements of downstream industry. The regenerated cellulose fiber prepared from *Arundo donax* L. Antibacterial activity about against *Escherichia coli*, *Staphylococcus aureus* and *Candida albicans*. In addition, *Arundo donax* L. cellulose fiber has demonstrated good antiviral performance against influenza A viruses H3N2 and H1N1. Therefore, *Arundo donax* L. fiber can be widely used in clothing, textile, medical supplies and other fields. The study above provides a basis for the development of *Arundo donax* L. resources.

## Figures and Tables

**Table 1:** *Arundo donax* L. chemical composition list.

	Types	Value
Ingredients		
holocellulose (%)		72.98 ± 2.80
*α*-cellulose (%)		55.2 ± 0.7
moisture (%)		4.56 ± 0.17
Benzene-alcohol extract (%)		1.69 ± 0.13
Lignin (%)		20.03 ± 0.14
Ash (%)		3.56 ± 0.16

**Table 2:** Comparison of detection indexes between *Arundo donax* L. pulp and others.

Types	*Arundo donax* L. Pulp	American COSMO Wood Pulp	South African (Broad-Leaved) Wood Pulp
*α*-cellulose (%)	91.35 ± 0.15	91.15 ± 0.35	94.25 ± 0.15
moisture content (%)	14.6 ± 0.2	7.2 ± 0.1	7.3 ± 0.1
Ash (%)	0.11 ± 0.01	0.05 ± 0.02	0.04 ± 0.01
Iron content (PPM)	13.0 ± 1.0	6.0 ± 1.0	5.5 ± 0.5
Cupric ammonia viscosity (mPa·S)	8.4 ± 0.1	23.1 ± 0.7	11.5 ± 0.4
Whiteness (%)	82.4 ± 0.4	93.8 ± 0.3	92.5 ± 0.5
Constant weight (g/m^2^)	798.5 ± 5.5	728.0 ± 7.0	1011.0 ± 7.0
Alkali absorption value(%)	607.0 ± 1.0	520.0 ± 2.0	539.5 ± 1.5
Middle big dust (one/kg)	1.5 ± 0.1	0.0 ± 0.0	0.0 ± 0.0
Little dust (mm^2^/kg)	74.0 ± 2.0	12.5 ± 0.5	13.0 ± 0.0
Pentose (%)	4.28 ± 0.15	2.72 ± 0.06	/
Reactivity (s)	15.5 ± 0.5	7.5 ± 0.5	/

(/: Not detected).

**Table 3:** Reagent ratios for biomass *Arundo donax* L. cellulose dissolution experiments.

Percentage of *Arundo donax* L. Pulp	Activation Time (h)	TH:DS	Defoaming Time (h)	Aromatic Polyoxyethylene Ether (mL/kg)
30~50%	2~3	1:9	8~10	(1~3)/(1~10)
30~50%	2~3	2:8	8~10	(1~3)/(1~10)
30~50%	2~3	3:7	8~10	(1~3)/(1~10)
30~50%	2~3	4:6	8~10	(1~3)/(1~10)
30~50%	2~3	5:5	8~10	(1~3)/(1~10)

**Table 4:** Antibacterial properties of biomass-regenerated cellulose fiber made from *Arundo donax* L.

Test Item	Standard Antibacterial Value	Actual Value	Effect Evaluation
*Escherichia coli* (ATCC 6538)	≥70%	>99%	with antimicrobial effect
*Staphylococcus aureus* (ATCC 10231)	≥70%	>99%	with antimicrobial effect
*Candida albicans* (8099)	≥60%	>99%	with antimicrobial effect

**Table 5:** Antiviral properties of biomass regenerated cellulose fiber from *Arundo donax* L.

Name of Experimental Virus	Antivirus Activity Value	Anti-Virus Activity Rate (%)	Effectiveness Evaluation
Influenza A virus H3N2 (ATCC VR-1679)	>4.85	99.99%	with antiviral effect
Influenza A virus H1N1 (ATCC VR-1469)	>5.05	99.99%	with antiviral effect

## Data Availability

Data are contained within the article.
